# Chemical Composition and Free Radical Content During Saharan Dust Episode in SE Poland

**DOI:** 10.3390/molecules30244799

**Published:** 2025-12-16

**Authors:** Bogumił Cieniek, Dariusz Płoch, Julia Brewka, Katarzyna Kluska, Ireneusz Stefaniuk, Idalia Kasprzyk

**Affiliations:** 1 Institute of Materials Engineering, University of Rzeszów, Pigonia 1, 35-939 Rzeszow, Poland; dploch@ur.edu.pl (D.P.); istefaniuk@ur.edu.pl (I.S.); 2 Faculty of Biotechnology, Collegium Medicum, University of Rzeszów, Pigonia 1, 35-939 Rzeszow, Poland; jbrewka@ur.edu.pl (J.B.); kborycka@ur.edu.pl (K.K.); ikasprzyk@ur.edu.pl (I.K.)

**Keywords:** free radicals, EPR, Saharan dust, particulate matter, air pollution

## Abstract

This study aimed to verify whether Saharan dust reached south-eastern Poland in spring 2025 and to assess its influence on the chemical composition and oxidative potential of particulate matter. Using an ultra-sensitive Dekati instrument, aerosols were measured across fifteen size fractions (6 nm–10 µm), enabling the detection of particulate matter even in the finest particles—a feature not previously documented for Saharan dust. Electron paramagnetic resonance spectroscopy was used to quantitatively determine and identify radicals associated with different PM fractions. The analysis revealed a high content of ultrafine particulate matter (<1 µm), which may pose a potential risk to human health. The chemical composition of the particulate matter confirmed the long-range transport of Saharan dust over SE Poland at the beginning of March 2025. EPR measurements indicated a relatively large amount of pollutants that exhibited magnetic properties, which were not detected in the control samples. The use of advanced measurement instrumentation enabled the detection of ultrafine fractions and the identification of free radicals associated with Saharan dust, providing new insight into its oxidative potential and chemical reactivity.

## 1. Introduction

Particulate matter (PM) is one of the key atmospheric pollutants that has a significant impact on the climate, public health, and the functioning of ecosystems. It is a complex, heterogeneous mixture of solid and liquid particles originating from both natural sources (e.g., erosion, volcanic emissions) and anthropogenic sources (e.g., fossil fuel combustion, transportation). The size range of suspended dust includes particles with diameters ranging from several nanometers to approximately 100 µm. The chemical composition of the particles is heterogeneous, depending on the source of emission and the time spent in the atmosphere. In addition, dust undergoes numerous physicochemical processes in the atmosphere, which lead to changes in its properties and reactivity [1,2]. The main components of particulate matter are carbonaceous material comprising organic and inorganic fractions together with elemental carbon, as well as mineral material containing trace elements, secondary inorganic aerosol, and water [3]. During the heating season, the concentration of particulate matter in the air in urban areas increases due to emissions from heating homes with fossil fuels, thereby changing its composition. Additionally, the organic fraction is increasing (organic and elemental carbon, hydrocarbons), as well as water-soluble ions and inorganic elements, including heavy metals and trace elements [1,4,5]. The proportions of the dust components also depend on local meteorological conditions and atmospheric phenomena, such as the influx of desert dust [1,2,3]. In recent decades, the frequency of desert dust influxes into Europe has increased, mainly from the Sahara, but also from regions of the Aral-Caspian and the Middle East. During these episodes, PM concentrations increase, often exceeding accepted standards, which has an adverse effect on human health and well-being. These phenomena occur most frequently in spring and summer, when thermal and dynamic conditions favor the transport of particles into the upper layers of the troposphere [6,7,8]. The influx of desert dust to Europe, particularly from the Sahara, is influenced by several key meteorological conditions such as the movement of hot air masses when there is a low-pressure system in North Africa, which facilitates the northward movement of dust-laden air, unusually high temperatures for the season, strong winds, particularly those associated with cyclonic activity or cold fronts [1,2,3,9]. The phenomenon is well documented mainly for Southern Europe, with Spain, France, and Italy being the countries most affected by Saharan dust, both in terms of concentration levels and frequency of occurrence [10]. Although Saharan dust usually reaches the Mediterranean basin, it is increasingly observed in Central and Northern Europe, where it is becoming a permanent feature of contemporary climatic conditions [6,7,8]. In 2016–2017, dust events with concentrations exceeding 1 μg/m^3^ occurred with varying frequency across Europe: 5–10% of the time in Ireland, Denmark, and northern Great Britain; 10–15% in northern France, northern Germany, and the Netherlands; and the highest frequency, 20–30%, in southern France, southern Germany, and the Czech Republic [10]. The first well-documented influx of Saharan dust into Poland occurred in October 2001. The dust originated in the Sahara and traveled through Great Britain and Scandinavia before reaching Central Europe [11]. In 2000–2002, the EARLINET network recorded more than 130 days with Saharan dust layers, mainly in southern and southeastern Europe, with some episodes reaching the northern and northeastern parts of the continent [12]. Subsequent episodes occurred in July 2013, when dust from Western Sahara reached Poland after a long route over the Atlantic and the British Isles [13], and on 29–30 June 2019, when models indicated a 5-day transport from North Africa. The most surprising case was the inflow in February 2021 over Warsaw (central Poland), the first well-confirmed winter episode [9]. On February 1–7 of the same year, Saharan dust was also recorded in another part of Poland, detected in snow [14]. The dust usually originated from the Sahara and was transported to Central Europe sometimes via unusual routes, e.g., through the mid-Atlantic and Western Europe or the British Isles, before reaching central and northern Europe [10,11,12,13].

The average size of Saharan dust particles ranges from 1 to 20 µm, with the fine fraction being the most persistent in the atmosphere and responsible for long-distance transport to Central and Northern Europe [6,7,8,15]. The chemical composition of the dust reflects both the mineralogical characteristics of the source areas and the atmospheric changes that occur during transport. In terms of minerals, dust is dominated by silicates, iron oxides, carbonates, and admixtures of sulfates and chlorides of marine or anthropogenic origin. Clay minerals, quartz, and calcite have also been detected, with their proportions varying depending on the season and the intensity of advection. As the transport distance increases, the proportion of coarse fractions decreases, and the importance of fine particles rich in iron oxides increases [6,7,8]. Analyses confirm the presence of elements such as Al, As, Ca, Co, Cr, Cu, Fe, K, Mg, Mn, Na, Ni, Si, Ti, Pb, and Zn, typical for mineral material of desert origin [14,15,16,17]. The high frequency and intensity of dust events in the Sahara are associated with real health risks, including increased hospitalizations for respiratory and cardiovascular diseases during dust storms [18] and a significant number of premature deaths attributed to exposure to particulate matter [10,19]. Exposure to Saharan dust causes irritation of the eyes, nose, and throat, as well as breathing difficulties, and can lead to lung diseases such as asthma and pulmonary fibrosis. In vitro studies have shown that dust increases the expression of granulocyte and macrophage growth factors, which are associated with the development of lung diseases [20]. In countries located close to the dust source, the main exposure factors are coarse particles, while at longer distances, finer particles predominate and can penetrate deeper into the human respiratory system [10]. Ultrafine particles (UFPs), smaller than 100 nanometers, are particularly dangerous to human health due to their ability to penetrate deep into the respiratory system and enter the bloodstream. They induce inflammation, irritation, and impaired lung function, and their presence is associated with increased mortality from respiratory diseases. Furthermore, UFPs substantially increase cancer risk, particularly the risk of lung cancer, as they can transport toxic and carcinogenic compounds, leading to cellular damage, oxidative stress, and chronic inflammation [21].

Desert dust is not a passive component of the atmosphere, but a chemically active carrier of substances with oxidative properties. Fine fractions (PM_2.5_ and smaller) are characterized by a particularly high ability to generate reactive oxygen species (ROS) due to the presence of soluble transition metals, especially iron, copper, and manganese. These metals catalyze redox reactions leading to the formation of free radicals, which can cause oxidative stress in the cells of living organisms. Desert dust also contains significant amounts of elemental and organic carbon, which promote the formation of environmentally persistent free radicals (EPFRs) [22,23,24,25]. These radicals can remain on the surface of particles for a long time, retaining high chemical activity even after being transported over long distances [15,26,27]. Studies indicate that EPFRs are stabilized on aluminosilicate mineral surfaces and iron-rich fractions, allowing them to remain reactive for days or even weeks [28]. Elevated oxidative potential of dust observed during dust storm events (suggests that the smallest particles may induce stronger inflammatory responses than coarser fractions [26]. These chemical and physical properties of desert dust have direct consequences for human health. The combination of its chemical composition and ability to generate reactive oxygen species (ROS) means that even short-term exposure can lead to the exacerbation of respiratory and cardiovascular diseases, and sometimes to death. These mechanisms involve oxidative stress induction, damage to cellular membranes, activation of macrophages and neutrophils, and increased production of proinflammatory cytokines, as shown in epidemiological studies. More recent research indicates that high levels of EPFRs correlate with increased hospitalization rates, particularly among elderly individuals and children [28]. The increase in the frequency and intensity of dust storms associated with climate change further increases the potential risk to people exposed to these dusts [18,19,26,28,29]. As a result, desert dust transport is becoming an increasingly important global public health concern.

Refs. [30,31,32] show there is still a lack of comprehensive studies that cover the full spectrum of particle sizes of Saharan dust. Most existing research focuses mainly on its geographical distribution and the PM_2.5_ and PM_10_ fractions [9,14,33,34]. Moreover, the literature does not provide data on the inflow of desert dust into south-eastern Poland. Therefore, when weather models indicated at the beginning of March 2025 the possibility of conditions favorable for the transport of air masses from the Sahara to Europe, air-quality monitoring was undertaken to fill this research gap and assess the extent of the phenomenon in the region.

The aim of this study was to verify whether Saharan dust actually reached south-eastern Poland during the analyzed episodes and to assess how its presence affected the chemical composition and physicochemical properties of suspended particulate matter. Although elevated concentrations of particulate matter were recorded during the study period and atmospheric transport models indicated the possible inflow of air masses from the Sahara, the national environmental inspectorate did not conclusively confirm that Saharan dust was the source of the observed increase. Therefore, this study sought to determine whether Saharan dust transport to SE Poland indeed occurred and, if so, how it influenced the composition and properties of atmospheric aerosols. To verify this hypothesis, an ultra-sensitive Dekati instrument was used to measure and analyze aerosols across fifteen size fractions, ranging from 6 nm to 10 µm. Particular attention was given to the identification of free radicals, which have been rarely investigated in the context of Saharan dust. Owing to the applied instrumentation, it was possible to detect their presence even in such fine fractions, which has not been previously documented. Based on existing evidence that minerals present in Saharan dust can catalyze redox reactions leading to the formation of reactive oxygen species [22,23,24,25], the hypothesis that Saharan dust transport affects the level and characteristics of free radicals was tested by comparing results with periods without dust advection. This study provides a comprehensive understanding of the relationships between particle size, chemical composition, and oxidative potential of Saharan dust, offering new insights into its role in atmospheric chemistry and its potential impact on human health.

## 2. Results and Discussion

### 2.1. Structural Properties

**Figure 1:**
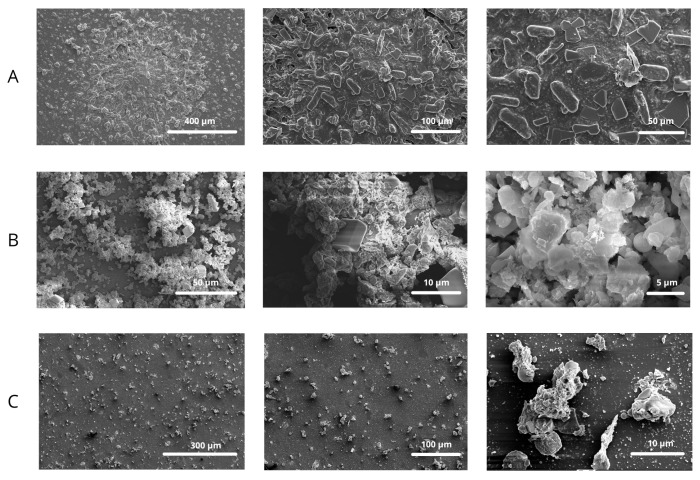
SEM images of PM deposited at filters #7 (**A**), #12 (**B**), and #14 (**C**).

**Figure 2:**
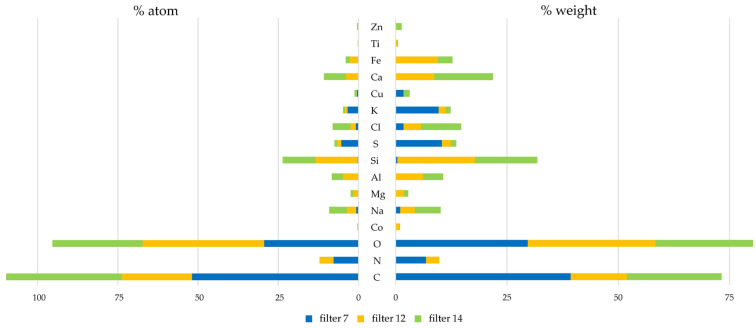
Atomic and weight percentages of elemental composition from filters #7, #12, and #14.

In order to characterize the morphological and chemical properties of PM, selected filters from the Dekati impactor were analyzed with a scanning electron microscope (SEM) (Figure 1) and energy-dispersive X-ray spectroscopy (EDX) (Table 1 and Figure 2). For detailed analysis, filters corresponding to the PM_2.5_ particle size range, as well as those exhibiting the highest EPR signal intensities, were selected. Filter #7 represents particles within the PM_2.5_ range, whereas filters #12 and #14 are characterized by distinctly elevated EPR signal intensities.

Based on the EDX analysis of filters collected on consecutive sampling days, a distinct pattern in the elemental composition of suspended particulate matter was observed (Table 1 and Figure 2). Filters #12 and #14 exhibit a high degree of similarity in elemental composition, along with a clear segregation of atoms between particle size fractions. Elements such as Co, Na, Mg, Al, Si, Ca, Fe, Ti, and Zn were detected on filters #12 and #14, but were absent or present only in trace amounts on filter #7. In contrast, C, O, S, Cl, and K were identified on all three filters (#7, #12, and #14). Nitrogen (N) was detected on filters #7 and #12, while copper (Cu) was observed on filters #7 and #14. The distribution of elements detected on individual filters closely correlates with the amount of pollutants; for example, sulfur is detected in the largest amount on filter #7. Similar results were obtained by Ainur et al., where sulfur compounds were found only for PM_10_ pollutants [35]. Overall, the data indicate that filter #7 represents a more carbonaceous, combustion-related fraction. The elemental composition identified on filters #12 and #14 is characteristic of mineral desert dust, with additional contributions from mixed-origin aerosols, and is consistent with findings reported by other authors investigating Saharan dust events [14,15,16,17,27]. Presence of the elements: Al, Ca, Co, Cu, Fe, K, Mg, Na, Si, Ti, and Zn, are typical for mineral materials of desert origin. The presence of the elements S, C, and N we associated with EPFR environmental pollutants, as confirmed by literature data [35,36,37]. The results confirm that, although air quality norms were not exceeded, Saharan dust was transported to Rzeszów, southeastern Poland. These findings indicate that the elemental composition of suspended particulate matter is strongly influenced by particle size, which may reflect variations in emission sources and physicochemical transformations occurring in the atmosphere [1,2,3,5]. Understanding these relationships is crucial for evaluating the effects of particle size on human health and the environment [18,19,29].

### 2.2. Dekati Measurements

**Figure 3:**
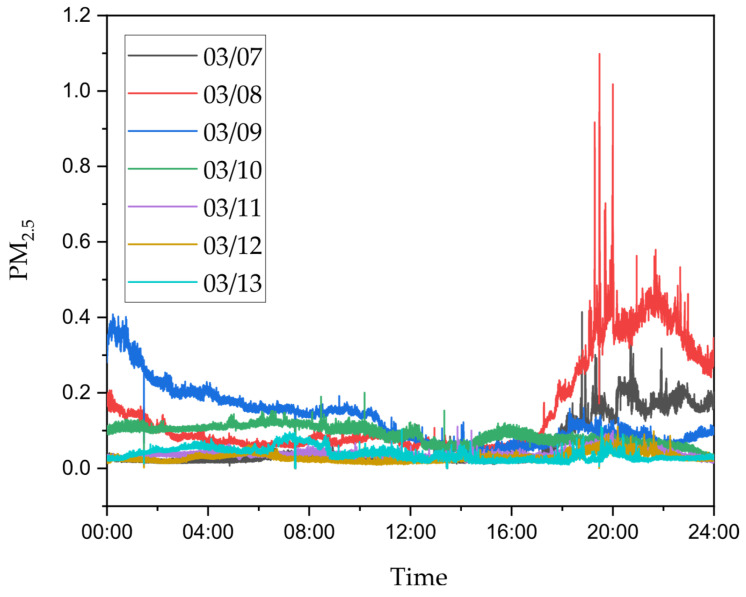
Changes in PM_2.5_ values depending on the time of day.

**Figure 4:**
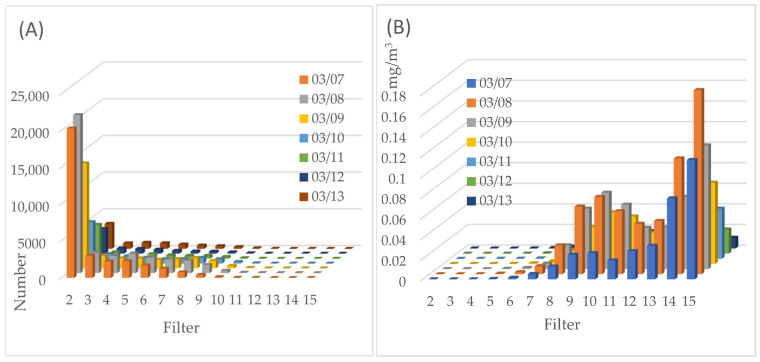
The number (**A**,**B**) mass of particulate matter (PM) collected on the impactor filters over consecutive sampling days.

Data obtained from the Dekati ELPI+ device were processed and analyzed to determine both the particle number concentration and the particle mass concentration for each impactor (Figure 3 and Figure 4). The analysis revealed clear differences in particle concentrations between the measurement periods.

The highest particle concentrations were recorded on March 8 (Figure 3, red line) and during the night between March 8 and 9 (Figure 3, blue line), coinciding with the period of Saharan dust transport over the region.

The smallest particles are the most numerous (impactor #2—0.0153 um), while most of the mass is concentrated in large particles. It is worth noting the local decrease in particle mass on filter #11 visible in the measurements from March 7 (Figure 4B), as well as from March 11–13. This corresponds to the days when there was no dust from the Sahara. At the same time, there is a local maximum in mass on March 08–10 on filters #9, #10, and #11, directly related to dust from the Sahara. It should be noted that the air pollution norms were not exceeded. At the same time, when comparing the quantity and mass of particles on March 8, when we observe the maximum, to the so-called “normal” level on March 12 and 13, we see a more than tenfold increase in the amount of pollution caused by dust from the Sahara.

### 2.3. EPR Spectroscopy

**Figure 5:**
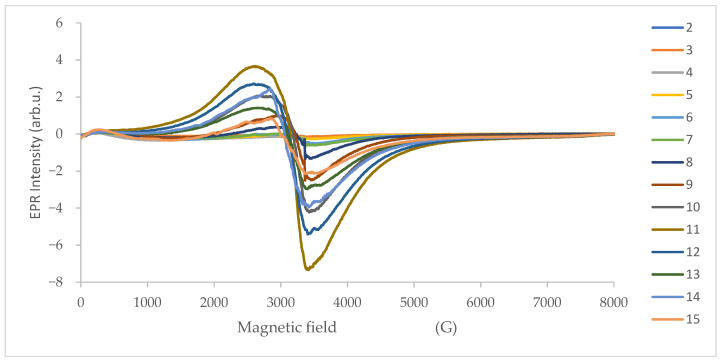
EPR spectra depending on the filter.

Filters with PMs obtained from the Dekati device were analyzed with EPR spectroscopy. Also, a clear filter without PM was measured, and no EPR signal was detected. Figure 5 shows the EPR spectra depending on the filter.

**Figure 6:**
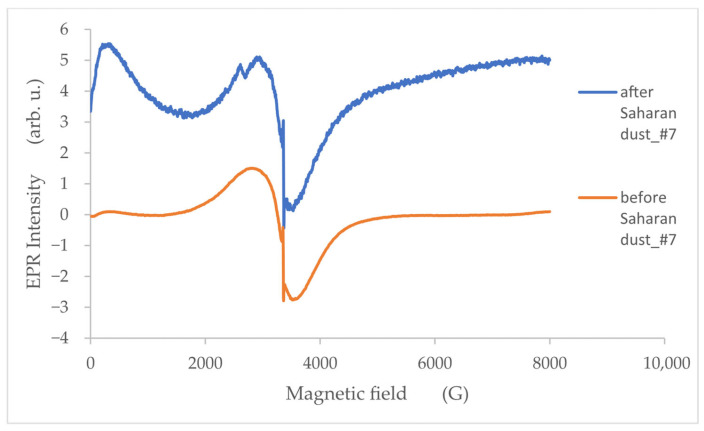
EPR spectra for filter #7 during the Saharan dust episode (blue line) and during the period without dust influence (orange line).

The shape and intensity of the EPR spectra obtained on 12–16 February 2005, representing the period without Saharan dust, were compared with the spectra recorded during the dust-affected episode [38]. Figure 6 shows a comparison of the EPR spectra from filter #7. For the spectra with Saharan dust, we can observe additional weak lines in the vicinity of 2500G. Moreover, the low-field magnetic absorption (LFMA) line, associated with magnetic interactions [39], appears to be significantly stronger.

When analyzing the obtained EPR spectra, a narrow line for g_eff_ = 2.0018 (effective g-factor) was observed, originating from carbon radicals and a broad line attributed to EPFRs of carbon-oxygen and oxygen radicals and other paramagnetic centers, mainly metal ions. (Fe, Cu) [25]. The g-factor is a useful parameter for identifying EPFR types [40,41]. Carbon-centered stable free radicals typically have a g-factor of below 2.003, carbon-centered radicals with an adjacent oxygen have a g-factor between 2.003 and 2.004 [42]. Stable oxygen-centered persistent radicals typically have a g-factor above 2.004. Smoke and tar produced by combustion of coal and petroleum contain high amounts of terpenoid free radicals with g-factors of approximately 2.0032 [43]. The g-factor values of the EPFRs particles in the atmosphere range from 2.0030 to 2.0047, which depend heavily on the chemical composition and source of the PMs [25,44,45].

**Figure 7:**
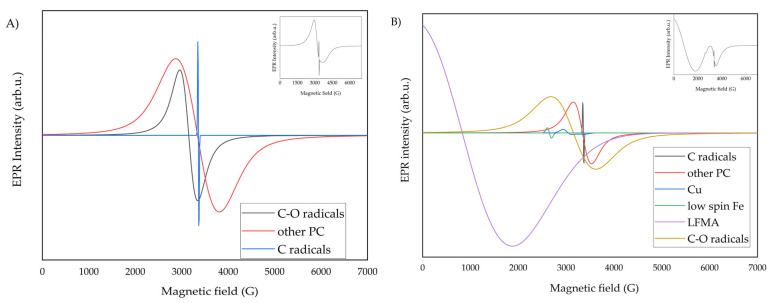
The EPR line split into components, (**A**) for filter #7 from February, and (**B**) from March with PM from the Sahara. The insets show the theoretical spectra as the sum of the components.

**Figure 8:**
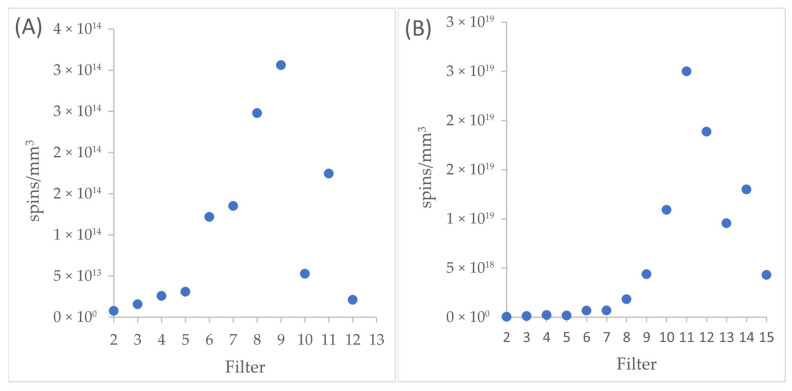
(**A**) spin values per mm^3^ and on the filter, calculated for a narrow line (carbon radicals), and (**B**) spin values per mm^3^ on the filter, calculated for a broad line (remaining radicals and paramagnetic centers).

The components of the entire EPR line were extracted using EasySpin software (version 6.0.11) [46]. The components are shown in Figure 7A for the February spectra and in Figure 8B for the spectra with Sahara dust.

EPR spectra from February consist of at least three lines, assigned to the carbon radical (g_eff_ = 2.0014), C-O radicals (g_eff_ = 2.016), and other paramagnetic centers (g_eff_ = 2.131). In Saharan dust, the EPR spectrum consists of at least 6 lines, 3 of which are consistent with the February spectra. Additionally, we assign lines originating from copper, from low-spin iron (ferriheme, g_eff_ = 2.537) [47], and the broad LFMA line, related to magnetic interactions (g_eff_ = 8.10883). This confirms the results presented by Szuszkiewicz et al. in 2023 [14], concerning the study of dust from the Sahara. The total number of spins was determined for a narrow line (carbon radicals, Figure 8A), and for a broad line (remaining radicals and paramagnetic centers, Figure 8B). The EPR method enables the detection of harmful EPFRs in air pollution, which are proportional to the mass of the samples. The quantity is measured by the number of EPFR spins per unit volume.

It should be noted that, depending on the type of radicals, the maximum is found at a different filter value. For carbon radicals at filter #9, and for other radicals and paramagnetic centers at filter #11. Comparing these maxima with the maxima of measurements taken in February [38], we observe a shift from #8 to #9, and from #12 to #11, for measurements with Saharan dust pollution. In addition, the nature of the EPR line is changing. For the February measurements, the shape of the EPR line was regular, similar to a Gaussian curve, while the current measurements show an irregular course with local minima. If we compare the EPR spectrum directly, we also observe differences. In summary, we observe clear differences in EPR measurements from the period with and without Saharan dust influx.

## 3. Materials and Methods

**Figure 9:**
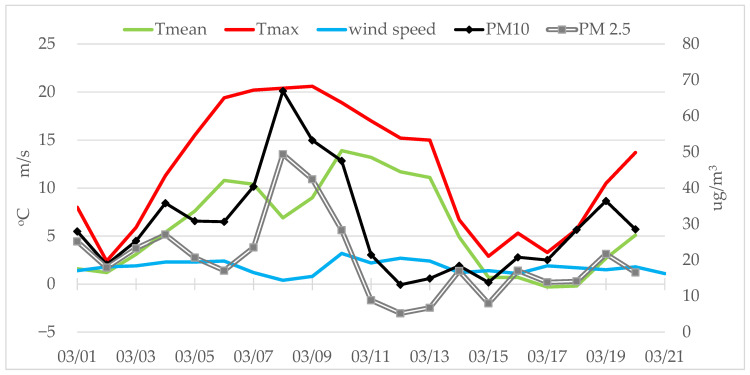
Daily variation of PM_10_ and PM_2.5_ concentrations in relation to meteorological parameters in March 2025, Rzeszów, Poland [49].

**Figure 10:**
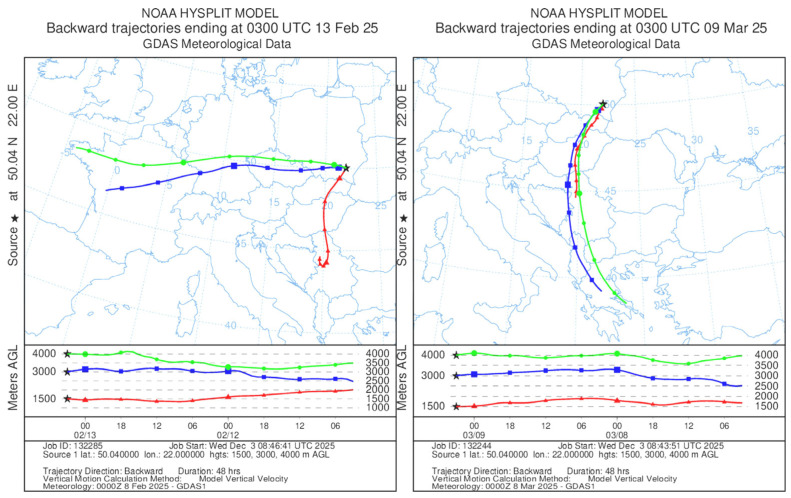
Backward trajectories with HYSPLIT mode for 13 February 2025 and 9 March 2025.

In Rzeszów, SE Poland, a comprehensive air-quality assessment was performed, beginning with control measurements in February 2025 to establish baseline conditions, and followed by the main sampling campaign conducted between 6 and 15 March 2025, aimed specifically at investigating the potential inflow of Saharan dust into the region. During the control measurements in February 2025, meteorological conditions, particularly air temperature, were consistent with the multi-year averages. In contrast, March 2025 was unusually warm, with an average temperature of 6.8 °C, exceeding the long-term average [48,49]. The lack of precipitation, weak winds, and the occurrence of thermal inversion contributed to an increase in the concentration of particulate matter originating from low emissions and the resuspension of pollutants (Figure 9). In addition, warm tropical air flowed into Europe, carrying Saharan dust with it (Figure 10). The Chief Inspectorate of Environmental Protection in Poland issued warnings of a possible inflow of Saharan dust over the country during the analyzed period. However, the final report did not confirm that the observed increase in particulate matter concentrations was directly associated with this episode [50].

**Figure 11:**
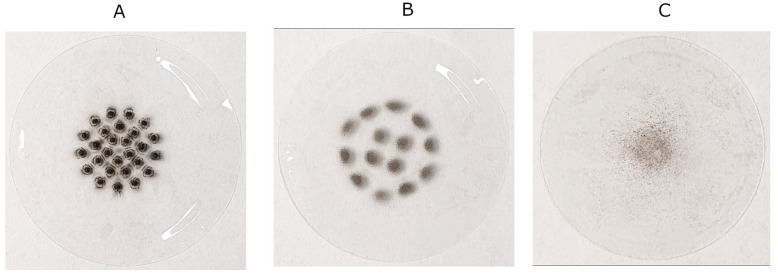
Photos of PM deposited at filters #7 (**A**), #12 (**B**), and #14 (**C**).

Samples of pollution from air were collected using a Dekati Electrical Low Pressure Impactor (ELPI+, Dekati Ltd., Kangasala, Finland) [31]. It is an advanced device designed to measure the number and size distribution of airborne particles [30]. The ELPI provides real-time measurement of particle concentration and size in a wide range from nano- to microscale, making it suitable for various applications, such as environmental research, industrial processes, and health-related studies [32]. The sampling flow rate was maintained at 10 L·min^−1^, and the instrument was calibrated according to the manufacturer’s specifications prior to each measurement campaign. ELPI is equipped with a cascade impactor, used to collect particles of selected sizes (see Table 2). It consists of two co-linear plates, the second of which has small holes. The plate with holes is a separating plate, while the other plate collects particles. The aerosol passes through the holes at high speed, then makes a sharp turn to pass between the plates. Particles larger than a certain size are unable to make this movement and settle on the collecting plate. We used polycarbonate filters to collect air pollutants on them (Figure 11). In ELPI, particles are charged in a corona charging system before entering the impactor. The charged particles are then segregated in the impactor. The current generated by the collection of particles on the collection plates is measured for each stage of the impactor. In this way, data is obtained in real time.

In the impactor, the phenomenon of diffusion causes particles with a diameter smaller than the cutoff diameter of the impactor stage to be collected by it anyway. The velocity of particles collected in the collection plate is zero, and the phenomenon of diffusion causes some of them to form a mesh of particles near the collection plate. This phenomenon occurs in all instruments that use an impactor. To overcome these losses, a correction algorithm is used for ELPI readings, as these losses are measured simultaneously with the efficiency curves for all stages. This algorithm is implemented in the ELPI+VI software (version 2.1). The algorithm takes into account both diffusion and spatial losses in the charging system. The correction method is described in more detail in Virtanen et al. [51]. The accuracy of measurement in ELPI depends on the size and type of particles being measured. The measurement directly relates to the current they carry, so the accuracy of particle measurement is actually the accuracy of current measurement. Sensitivity is better for larger particles, as the current intensity is significantly greater for them than for small particles. ELPI can operate in four measurement ranges: 10,000 fA, 40,000 fA, 100,000 fA, and 400,000 fA. When using smaller measurement ranges, the level of interference is lower, and in the case of larger ranges, it is higher. Therefore, measurement accuracy also depends on the measurement range. Interference levels, and thus measurement accuracy, are also affected by factors such as sudden temperature changes or instrument movements.

The electrical currents detected at each impaction stage were continuously recorded and converted to particle number concentrations using the Dekati ELPI software (version ELPI+VI 2.1) [31,52]. The resulting data were further processed to obtain particle number and mass size distributions, which were subsequently analyzed in relation to meteorological parameters. Since the Dekati ELPI device operated continuously, with a measurement rate of 1 s, the RAW data consists of 86,400 measurement points per day, simultaneously for all filters. The relative difference between the measured current and the current corrected for size range and diffusion losses is less than 4%. The filters were not replaced during the measurement period, and the dust that settled on each of them is the sum of the entire study period.

Filters were then microscopically analyzed using a high-resolution scanning electron microscope SEM Helios 650 Nanolab manufactured by FEI (Hillsboro, OR, USA). The chemical composition of PM deposited on the filters was determined using an EDX Apollo X energy dispersive spectrometer from EDAX. The tested samples were coated with an ultrathin layer of gold to eliminate electrostatic charging. Surface imaging was performed using secondary electrons (SE) at an accelerating voltage of 5 kV. Filters were also analyzed in an electron paramagnetic (EPR) spectrometer. EPR measurements were performed on X-band (ν ≈ 9.4 GHz) with modulation of the magnetic field at 100 kHz, by a Bruker multifrequency and multiresonance FT-EPR ELEXSYS E580 spectrometer (Rheinstetten, Germany) with a sensitivity of 7 × 10^9^ spins per 0.1 mT, having a resolution of 2.35 (micro) T or even better [53]. Components of the EPR line were extracted using EasySpin software (version 6.0.11) [46]. Samples collected in March 2025 were analyzed in comparison with reference material obtained in February 2025, representing a period without episodes of Saharan air mass transport over Europe.

## 4. Conclusions

The results of this study provide clear evidence that Saharan dust reached south-eastern Poland during the analyzed period, even though air quality threshold values were not exceeded. The presence of Saharan dust particles in the air was confirmed through the application of highly sensitive measurement techniques and a detailed analysis of particle size distributions and EPR spectra. The analysis revealed a high number of ultrafine pollutants (<1 µm), which may pose a potential health risk. EPR measurements showed a relatively large amount of pollutants with magnetic properties, which were not observed in the control sample, but are characteristic of Saharan dust. Also, the distribution of spin values per mm^3^ showed a clear variation in relation to the control sample. A similar trend of atomic segregation by filter fraction was observed in both EDX and EPR analyses.

The obtained findings highlight that conventional air quality monitoring systems, based primarily on routine PM_2.5_ and PM_10_ measurements, are not capable of detecting such subtle episodes of long-range dust transport. As a result, the information they provide may be incomplete and may not accurately reflect the true scale of exposure or potential risks to human health and ecosystems. The use of advanced instrumentation enabled the detection of ultrafine fractions and the identification of free radicals associated with Saharan dust, offering new insight into its oxidative potential and chemical reactivity. This emphasizes the importance of integrating high-resolution and chemically sensitive analytical techniques into air monitoring networks to better assess the real impact of long-range dust transport on air quality and environmental health. The use of the Dekati instrument in combination with the EPR method brings a new quality to air pollution research.

## Figures and Tables

**Table 1:** Percentage weight and atomic content of the elements composition from EDX, for filters #7, #12, and #14.

% Weight	C	N	O	Co	Na	Mg	Al	Si	S	Cl	K	Cu	Ca	Fe	Ti	Zn
filter #7	39.35	6.83	29.67	0.00	1.04	0.00	0.08	0.40	10.40	1.78	9.67	1.79	0.00	0.00	0.00	0.00
filter #12	12.65	2.99	28.71	0.85	3.27	1.91	6.14	17.42	2.00	3.90	1.60	0.00	8.69	9.49	0.39	0.00
filter #14	21.23	0.00	21.89	0.12	5.81	0.93	4.43	14.00	1.22	9.06	1.05	1.35	13.19	3.29	0.12	1.35
% atom	C	N	O	Co	Na	Mg	Al	Si	S	Cl	K	Cu	Ca	Fe	Ti	Zn
filter #7	51.88	7.76	29.39	0.00	0.71	0.00	0.05	0.22	5.35	0.79	3.40	0.44	0.00	0.00	0.00	0.00
filter #12	21.94	4.34	37.91	0.30	2.96	1.66	4.84	13.18	1.35	1.75	0.87	0.00	4.00	2.77	0.18	0.00
filter #14	36.00	0.00	28.11	0.04	5.46	0.78	3.41	10.23	0.79	5.50	0.54	0.76	6.73	1.19	0.05	0.45

**Table 2:** Filter number and corresponding particle size range in the Dekati impactor (D50% represents the aerodynamic diameter of each stage).

Impactor	1	2	3	4	5	6	7	8	9	10	11	12	13	14	15
D50% (µm)	0.006	0.0153	0.0303	0.0544	0.0949	0.155	0.257	0.384	0.606	0.953	1.64	2.48	3.67	5.4	9.94
Filter #	-	2	3	4	5	6	7	8	9	0	11	12	13	14	15

## Data Availability

Data available in a publicly accessible repository. The original data presented in the study are openly available in Repozytorium Danych Badawczych Uniwersytetu Rzeszowskiego, at https://rdb.ur.edu.pl/items/40460a77-c1aa-45d5-bff9-09fa727217a1 (accessed on 13 December 2025).
